# Anticipating governance challenges in synthetic biology: Insights from biosynthetic menthol

**DOI:** 10.1016/j.techfore.2018.11.020

**Published:** 2019-02

**Authors:** Barbara Ribeiro, Philip Shapira

**Affiliations:** a Manchester Institute of Innovation Research, Alliance Manchester Business School, University of Manchester, Manchester M13 9PL, United Kingdom; b Manchester Synthetic Biology Research Centre for Fine and Speciality Chemicals, Manchester Institute of Biotechnology, University of Manchester, Manchester M1 7DN, United Kingdom; c School of Public Policy, Georgia Institute of Technology, Atlanta, GA 30332-0345, United States of America

**Keywords:** Sociotechnical transition, Anticipatory governance, Emerging technologies, Responsible research and innovation, Synthetic biology, Menthol

## Abstract

This paper advances an anticipatory governance framework to investigate and prepare for the potential implications of an emerging technology. Within the growing domain of synthetic biology, we draw on an end-to-end assessment of biosynthetic menthol that incorporates consideration of multiple dimensions of production and use. Based on documentary analysis, available data, and interviews, our approach unfolds in three steps. First, we map the sociotechnical transition in menthol production, comparing existing agricultural and chemical production methods with new biosynthetic processes – or what we call the biological (bio) turn. Second, we explore the rationales, promises and expectations of menthol's bio-turn and explore the drivers of transition so as to clarify which goals and values innovation is addressing. Third, we reflect on the opportunities and challenges of such a transition to put forward an agenda for responsible innovation and anticipatory governance. The bio-turn in menthol is analysed through five responsible innovation dimensions: the potential distribution of benefits and burdens; social resilience; environmental sustainability; infrastructure and business models; and public perception and public interest. We consider the implications of our analysis both for the responsible development and application of synthetic biology for menthol and for the broader assessment and sociotechnical construction of emerging technologies.

## Highlights

• A transition to synthetic biology poses societal and environmental challenges.
• Anticipatory governance efforts can benefit from a focus on real-world applications.
• Tailored case-by-case approaches suggest that existing regulations may not suffice.

## 1 Introduction

Prominence has been given in recent years to the importance of building capabilities for the anticipatory governance of emerging technologies, pursuing responsible research and innovation (RRI) and situating dialogue about emerging technological innovations in the context of deliberations about societal futures (Grunwald, 2017; Guston, 2014; Stilgoe et al., 2013). These calls follow a longstanding and central feature of the assessment and evaluation of emerging technologies, which can be summarised as the consideration of both favourable and adverse potential implications of applications of that technology so as to inform public discussion, management and policy decision making (Coates, 1976; Porter et al., 1980; Rip, 1995; Schot and Rip, 1996).

Each promised emerging technology has its own distinct characteristics, applications and framing and is (or could be) associated with a particular mix of potential implications (Davidson, 2002). The trajectories of emerging technologies are also socially as well as economically shaped through interactions among varied public and private sector actors (Winner, 1980; MacKenzie and Wajcman, 1985; Williams and Edge, 1996). While some emerging technologies may be entirely novel, in many cases, there are already existing incumbent alternatives against which the relative advantages and disadvantages of emerging technologies can be compared. These particularities in the nature, moulding, and unfolding of emerging technologies reinforce arguments for ongoing, disaggregated and grounded approaches to assessment. Moreover, in each round of development, emerging technologies must be assessed in frameworks of current and anticipated economic, societal and environmental challenges including (but not limited to) competitiveness and globalization, inclusive and participatory development, and sustainability.

This paper contributes to a move towards more grounded approaches to anticipation and assessment for the governance of emerging technologies. It builds on a study conducted as part of a multi-year RRI project in which social scientists, embedded in a research setting (Le Feuvre et al., 2016), investigate the potential implications of the emerging field of synthetic biology. The study provides an in-depth analysis of a sociotechnical transition from conventional to biosynthetic methods for the production of a ubiquitous compound to explore the question of what are the areas of research, innovation, regulation and policy that should be prioritised for the responsible development and governance of synthetic biology. The importance of going beyond broad high-level category assessments of emerging technologies to add focus on specific applications and their diverse impacts is certainly evident in the emerging domain of synthetic biology. As defined by the National Academies of Science (2013), synthetic biology is “an emerging discipline that combines both scientific and engineering approaches to the study and manipulation of biology.” Also known as engineering biology, synthetic biology involves redesigning biological components and systems found in nature or making new ones from scratch, with a large array of applications promised in agriculture, chemicals, food, energy, environment, materials and medicine (Li and Shapira, 2015, see also RAE, 2009; König et al., 2013; Shapira et al., 2017).

Synthetic biology seeks to develop, replicate and optimise complex metabolic pathways that through evolution have allowed living beings such as plants to synthesize a series of compounds which are useful to not only these organisms themselves, but also to human societies. Frequently, synthetic biology is put forward as contributing to a global bioeconomy “project” that aims to foster economic activities based on renewable biological resources to produce food, energy, materials, and other products (EC, 2012). Biosynthetic products are located in the higher-value end of the bioeconomy spectrum, which includes a series of fine and speciality chemicals. While often thought to be indistinguishable from extant industrial biotechnology approaches, some supporters of the field argue that one of the distinct features of synthetic biology is its strong orientation towards “applications” (Eils et al., 2015), i.e. advancing the translation of research into products. This is pursued through concerted efforts from different disciplines within biological and computational sciences and engineering. Promises associated with this multidisciplinary field of research are wide-ranging and include meeting societal challenges through the sustainable production of, for example, anticancer compounds, essential vitamins, more resistant crops and advanced biofuels (Clarke and Kitney, 2016; Wurtzel and Kutchan, 2016).

One important area of focus of translational research in synthetic biology is flavours and fragrances to be used in food, cosmetics and the pharmaceuticals industries. This field of application focuses on addressing challenges related to seasonal variations in the supply of key compounds that are naturally produced by plants, and to provide consumers with produces that are potentially cheaper, higher in quality and more environmentally sustainable (Jullesson et al., 2015). However pervasive these applications may become in our everyday lives, analyses of the implications and the potential negative impacts of synthetic biology have been mostly directed towards the biomedical sector (e.g. risk to human health), the environmental risk posed by GM organisms, issues of security due to bioterrorism, and ethical debates around the creation of life (e.g. Anderson et al., 2012; Carr, 2011; Dana et al., 2012; SCENIHR, 2015). Other accounts have also considered the political economy of synthetic biology and what could be the wider implications for developing economies (e.g. Wellhausen and Mukunda, 2009).

Despite being lauded as an application-driven platform, there has been limited work to date on the implications of synthetic biology across its broad range of uses, motivating calls for an application-oriented turn in the social studies of the field and a focus on its real-world dimension (Marris and Rose, 2012; Schyfter and Calvert, 2015). This paper aims to contribute to this line of social enquiry by offering a focused prospective analysis of a specific application in the field of synthetic biology. We do this by investigating a sociotechnical transition in the production of menthol, an important component of a range of nondurable, fast-moving consumer goods, such as cosmetics, toothpastes, sweets, chewing gums and cigarettes (Trasarti et al., 2004).

Menthol is one of the world's most widely-used flavours and fragrances by volume. About 70% of the global supply of menthol is derived from plants (mostly *Mentha arvensis* or cornmint), with about four-fifths of the world's natural mint oil and menthol grown by farmers working at subsistence levels in India (Lange, 2015; Tiwari, 2016). The other 30% of global menthol production comprises compounds produced using chemical synthetic or semi-synthetic methods, with the market led by a set of large multi-national chemical companies (Lange, 2015). Menthol is now a target for research and innovation in the field of synthetic biology (e.g. Currin et al., 2018; Toogood et al., 2015) with the aim of deploying alternatives to conventional production methods (i.e. agriculture-based natural menthol or chemically-synthesized menthol). We denote this anticipated transition from conventional to synthetic biology methods as a “biological turn” (or “bio-turn”) in the production of the compound (i.e. biosynthetic menthol).

Based on a documentary analysis of reports and research papers in synthetic biology and menthol, as well as interviews with scientists working in the field of synthetic biology in the United Kingdom, our approach for exploring this bio-turn in menthol production is comprised of three steps. First, we map the sociotechnical transition in menthol production, with attention to agricultural, chemical and biosynthetic production processes. Second, we examine the background narrative of the bio-turn in menthol production through unpacking the rationale and objectives, promises and expectations behind the different production pathways. This allows us, in a third step, to explore the fundamental drivers of transition and to probe the goals and values that innovation is seeking to address (Bozeman and Sarewitz, 2011). These three steps are important as they add granularity to the case of synthetic biology. This is essential for appreciating the nuances of the real-world applications of relatively abstract, future-oriented developments in science, technology and innovation. Without a rich picture that connects synthetic biology to specific places, historical events, expectations, uncertainties and, importantly, the technical and material aspects of transitions, an attempt to draw an agenda for action would be largely speculative. Drawing on a thematic analysis of the sociotechnical transition in menthol production and the mainstream narrative on the opportunities and challenges of biosynthetic menthol, we extract a set of issues that are put forward as an agenda that can inform the anticipatory governance of synthetic biology in the field of flavours and fragrances. This agenda adds to the current RRI debate on the governance of emerging technologies by highlighting overlooked aspects in RRI analyses of emerging technologies and contributes to shedding light on the potential future implications of synthetic biology applications in everyday products.

## 2 Anticipatory governance in the context of synthetic biology

Our analysis is situated in the context of the anticipatory governance of synthetic biology and a broader end-to-end assessment of biosynthetic menthol that focuses on both the upstream and downstream dimensions of menthol production in relation to different actors and sectors. The end-to-end assessment is a type of approach related to real-time technology appraisal (Robert et al., 2013). In this paper, we build on an end-to-end assessment of the menthol case (see also Meckin et al., 2016; Meckin and Shapira, 2017). Anticipatory governance is a concept mobilized in the context of the management of emerging technologies in their early stages of development, i.e. while they are still amenable to management, through the use of such mechanisms as foresight, engagement, integration, feedback, and adaptation (Quay, 2010; Guston, 2014). The concept has been defined as “the cultivation of a societal capacity for foresight”, both as formal and informal, forward-looking and engagement-oriented practices (Barben et al., 2008). Anticipatory governance has been especially employed in social studies of nanotechnology (e.g. Shapira et al., 2015) and we argue that synthetic biology could also benefit from further social analysis through its lens.

Efforts to advance anticipatory governance are often mobilized in conjunction with initiatives to promote RRI (Ribeiro et al., 2017). Anticipatory governance builds upon transdisciplinary capacities to engage with actors across different disciplines and outside research circles; to critically reflect on our own assumptions and to explore multiple scenarios of technology development before research gets translated into applications (Guston, 2014; Karinen and Guston, 2010). Anticipatory governance therefore emphasises the importance of collectively reflecting on the environmental and social implications of emerging technologies and of building capacity to respond to unintended consequences, rather than predicting outcomes of technology implementation (Guston, 2014). Here, the main argument for favouring an ex ante approach to evaluation and deliberation in science and technology development rests on a longstanding paradigm in technology assessment, known as the Collingridge dilemma or the dilemma of control. Simply put, technological change becomes less reversible as technologies become embedded in society and as they develop towards larger sociotechnical systems. Given that these systems at the same time are shaped by and shape the society and the environment, our capacity to manage the consequences of technological change is likely to decrease with time (Liebert and Schmidt, 2010). Although Collingridge's dilemma of control needs to be complemented by more critical approaches to understanding social shaping of technology (Ribeiro et al., 2018), it does provide a compelling justification for the anticipatory governance of emerging technologies.

One of the very few studies that focus on the articulation of anticipatory governance in the context of synthetic biology is Wiek et al. (2012). Building on a critique of how visions and imaginaries around sustainability can sometimes be narrowly framed in synthetic biology discourse, and borrowing a model from nanotechnology studies, the authors offer a framework for addressing sustainability issues in the field. In their work, anticipatory governance dimensions are combined with those of transformational sustainability science to deliver a generic list of research activities, including the three main elements of anticipatory governance, according to Barben et al. (2008): foresight, engagement and integration of natural sciences and engineering with social sciences and humanities. However useful and translatable to synthetic biology the approach and the research questions put forward by Wiek et al. (2012) may be, we believe there is value in looking at specific, real-world applications of synthetic biology in order to grasp governance issues in more detail. Broadly speaking, the notion of anticipatory governance supports innovation models that aim to be more reflective, inclusive and deliberative. Reflection is a crucial capability relevant to discussions around responsibility – precisely that of carefully considering one's own beliefs and assumptions (Lynch, 2000) and being open to review and potentially change our own opinions when considering those of others (Dahlberg, 2004). As for inclusiveness and deliberation, these are dimensions that challenge historical processes in the governance of science, technology and innovation, which have narrowly involved more powerful actors such as experts, industry representatives and decision-makers, while excluding wider publics. Within a technocratic paradigm and through top-down governance tools, either decisions are made with no further input from other groups of actors, or the input of these actors does not have an impact on these decisions (Jasanoff, 2003; Thorpe, 2008). More inclusive and deliberative models of innovation should therefore be open to the values and concerns from different groups.

To grasp the potential broader issues that the development of emerging technologies such as synthetic biology might entail, first it is necessary to carefully examine the sociotechnical transition that takes place in the background of developments such as new processes for producing ubiquitous, everyday compounds such as menthol. The next section situates menthol within this shifting landscape in which different pathways of production might co-exist and potentially replace each other. We argue that it is important to understand in detail the specificities of different production pathways and value chains, as these shape the implications of a transition from conventional ways of producing a certain compound to new ones (e.g. Ribeiro and Quintanilla, 2015). As we hope to show, the feasibility and desirability of a transition in menthol production will depend on various considerations, which in turn embed specific values and concerns. These are later discussed in the paper as an initial step towards the anticipatory governance of this class of synthetic biology developments.

## 3 A sociotechnical transition in menthol production

Menthol is a “top-selling” flavour (Behr and Johnen, 2009), traditionally obtained from mint plants such as cornmint, spearmint and peppermint, which have been used for many centuries across the world in the production of popular flavours, fragrances and medicines. These common species names are used to refer to the well-known flavour of cooling effect menthol (Tucker, 2007). Besides menthol, a number of other aroma chemicals such as menthone, carvone, and linalool can be obtained from essential mint oil for use in food products, cosmetics, beverages and other industries (Verma et al., 2010). Cornmint (*M. arvensis* or *M. canadensis*) is also known as menthol mint, given its higher menthol content in comparison with other species (see Verma et al., 2010), and is therefore one of the species with higher economic importance in the menthol industry, along with spearmint (*M. spicata*) and the hybrid peppermint (*M. piperita*) (Tucker, 2007).

Menthol can be obtained through conventional pathways, such as by isolation from cornmint oil by distillation, followed by crystallization at low temperatures (i.e. natural menthol), and by partial or total synthesis (i.e. chemical or synthetic menthol) using different starting materials, including precursors obtained from crude oil, but also thymol from thyme oil or myrcene, a compound found in many plants, such as bay, lemon grass and wild thyme (Behr and Johnen, 2009; Lawrence and Hopp, 2007). In more recent developments in the field of synthetic biology, menthol has been produced in the laboratory using genetically modified bacteria (i.e. biosynthetic menthol) (Toogood et al., 2015). This section summarises the different ways menthol can be obtained to illustrate a transition from conventional (i.e. natural menthol and synthetic menthol) to biosynthetic routes.

### 3.1 Conventional menthol production

Although mint oils are produced in many parts of the world, including countries such as Argentina, Brazil, France, Thailand and the US (Verma et al., 2010), India and China are the two largest producers of “natural” menthol, obtained mostly from mint oil extracted from cornmint plants (Lawrence and Hopp, 2007). In the past, different countries dominated mint oil and natural menthol production. Japan, for example, was the main producer of mint oil from cornmint (a species introduced in Japan from China) in the first half of the 20th century, providing roughly 70% of the global demand. The Japanese experience was exported to Brazil in the early 1920s when a large number of Japanese emigrants arrived and settled in the country. Cornmint was mostly grown in southwest Brazil close to the border with Paraguay, which was also an important producer of mint oil at the time. Although Brazil was once the main global supplier of menthol, in the mid 1960's the sector could not cope with depressed prices of mint oil and the emergent competition with Indian cornmint oil, which is currently imported by both Brazil and Japan (Lawrence and Hopp, 2007).

India took over as a dominant global supplier of natural mint oil from China, which was previously the largest mint and menthol producer between the 1980s and 1990s, with smallholders growing cornmint in Eastern Central provinces. Currently, India is responsible for supplying about 80% of the global demand for natural mint oil and menthol (Lange, 2015; Tiwari, 2016). Although a major exporter, India's domestic consumption of mint accounts for around 40% of the total world consumption, followed by China (20%) and the EU (15%, with Germany and The Netherlands as main importers); the US counts for approximately another 15% of total global consumption.^1^ Cornmint plants were first introduced in India from Japan in the 1950's, in the Northern region of Uttar Pradesh. Since then, the Indian government, in collaboration with research institutes, has been optimising production through hybridisation and selecting cultivars of cornmint with higher menthol content. Along the years, India has dedicated considerable efforts to sustain and improve the cultivation of mint plants and the production of natural menthol, including genetic manipulation to improve the biosynthesis of mint oil and increase yields (Bose et al., 2013; Tiwari, 2016).

Mint fields in India are distributed over 163,000 ha of agricultural land dedicated to growing most of its cornmint in the regions of Uttar Pradesh, Haryana and Punjab (Verma et al., 2010). As in China, small-scale farmers (<1.5 ha of land) lead mint oil production in the country, which also counts with several crystallization factories for menthol production (Srivastava et al., 2002; Lawrence and Hopp, 2007). The large majority of mint growers are impoverished and farming systems are typically sustained by the work of different members of the same family (Singh et al., 2012). The menthol market in India is estimated to sustain the livelihoods of around 15 million people, with the price paid to farmers for the mint oil produced depending on its menthol concentration, i.e. higher percentages of menthol ensure higher prices (Bajaj, 2008; IFEAT, 2014). The price of natural menthol is very volatile as yields largely depend on environmental conditions (Hussain et al., 2010), but also market conditions, including pressures from the competition with synthetic menthol. Grey sources point out to a price of around $16.5/kg of natural menthol for 2015,^2^ while others have reported prices as low as $5/kg for Indian menthol in 2013.^3^

In recent years, the synthetic production of menthol has increased to meet the growing demand from the food and flavours industry as a solution to price volatility, uncertainty in supply and rising production costs of natural menthol (Etzold et al., 2009). Already in 1998, synthetic menthol represented 20% of the global production, aimed to obtain menthol from more reliable, alternative raw materials (Trasarti et al., 2004). At a competitive price varying between $15 and 20/kg, about 30% of the global production of menthol today corresponds to synthetic methods (Lange, 2015). Symrise, Takasago and, more recently, BASF are the three chemical companies currently leading the market (Lange, 2015).

Although supporters of natural menthol argue that its synthetic form has a lower quality of flavour when compared to the former (Tiwari, 2016), some argue that the key differential of synthetic over natural menthol is its economic strength and reliability given the ready availability of cheaper precursor compounds, sometimes in the country of production (Sell, 2003). These precursors include chemicals such as m-cresol, a compound obtained from petrochemical sources, used in the Haarmann and Reimer process adopted by the multinational company Symrise. Citral is the preferred precursor used by one of the largest companies of the global chemical industry, BASF, which obtains the raw material from isobutene and formaldehyde, sourced from crude oil (Schäfer, 2013; Parker et al., 2016). Another important producer of semi-synthetic menthol is Takasago, which uses myrcene as a raw material, a major compound of turpentine, found in pine resin (Behr and Johnen, 2009). These production processes are representative of semi-synthetic (Takasago) or synthetic (Symrise and BASF) routes, through which menthol is obtained from field-grown material or from fossil sources, respectively. While the semi-synthetic pathway is criticised for depending on the availability of natural compounds produced by plants that work as precursors in the biosynthesis of menthol (similarly to natural menthol), synthetic pathways are associated with less environmentally friendly processes, given their reliance on petrochemicals (Roberts, 2007).

### 3.2 Biosynthetic menthol production

A key concept in the biosynthetic production of fine chemicals is that of platform strains – an intermediary component of biosynthetic pathways, which contains the desired traits to be expressed in an organism, such as stress tolerance, fermentation, performance and substrate utilisation (Jullesson et al., 2015). The idea behind the concept is that of allowing microbial platforms to produce as many different target compounds as possible in a modular fashion through an extensive and accessible library (Chang and Keasling, 2006; Leferink et al., 2016). This process is facilitated through computational tools and, increasingly, by the use of automation, robotics and artificial intelligence methods such as machine learning (Bedbrook et al., 2017). Given the economic importance of the flavours and fragrances sector, a range of compounds has been the focus of research in synthetic biology, with many developments being undertaken in partnership with the industry. Vanilla, arguably one of the most important flavour compounds used as an additive in a variety of products, is a great example of earlier and current efforts in the design of de novo biosynthetic pathways in yeast (Hansen et al., 2009). Similarly to trends observed for menthol, the production of vanillin has gradually shifted from natural or field-grown vanilla pods to synthetically produced vanillin, with growing interest from synthetic biology. Today, synthetic vanillin it is sold at a much lower price compared to the natural competitor, covering approximately 99% of the demand for the product (Hansen et al., 2009).

Although the prices for natural menthol are not as prohibitive as those for natural vanillin, menthol has a global demand that sits today at roughly 30,000 t per year, and an economic importance close to that of vanillin and citrus (Kamatou et al., 2013). This is why menthol has also become a focus of synthetic biology. Recently, scientists have proven the technical feasibility of its production in the laboratory using recombinant bacteria (*E. coli*) (Toogood et al., 2015). Here, *E. coli* incorporates genes from *N. tabacum* (tobacco plant) and *M. piperita* (peppermint) that allow the biosynthesis of menthol, replicating the pathway existent in peppermint. Since chemical compounds like menthol can be toxic to host microorganisms such as bacteria, extracts of the recombinant *E. coli* were used for in vitro menthol production, which is different from using the organism itself as a production site. Several optimisation strategies followed to allow selection of the best host strain and understanding side reactions; these aimed at increasing the productivity of the process and the quality (i.e. purity) of the final product (Toogood et al., 2015). This route has only been demonstrated as a proof-of-concept, for which the technical and economic feasibility of larger-scale processes are yet to be evaluated.

As of today, research and patent activity focused exclusively on biosynthetic menthol is still in its infancy.^4^ Besides work by Toogood et al. (2015) developed in the UK, another relevant example includes work by Kim et al. (2015) from South Korea on myrcene as a starting material for menthol production. In 2013 and 2015, in collaboration with the National University of Singapore, the Massachusetts Institute of Technology (MIT) was granted the first and only two patents on the microbial engineering of chemical and pharmaceutical products with a focus on terpenoids, but explicitly mentioning menthol as a product of interest. However, the microbial production of terpenoids in general has been of longstanding interest in industrial biotechnology for at least two decades (Zhou et al., 2016), with around 200 original research papers published in the last five years and 11 patents since 2011.^5^ Some compounds can already be found in the market produced via microbial cell factory approaches by companies such as Amyris (US) and Evolva (Switzerland), who focus on the production of orange and woody tastes and odours from yeast (Schempp et al., 2018). Meanwhile, a range of biotechnological and fermentation players from the sector have been considering the microbial production of menthol as one of many terpenoids of interest (Leffingwell, 2015).

The technical and commercial viability of the large-scale microbial production of flavours and fragrances, besides issues around consumer perception, market preference and regulatory aspects (e.g. risk assessment, labelling) remain hotly debated aspects of biosynthetic production of menthol and other compounds (see Marris, 2015; Baumann, 2016; Epstein and Vermeire, 2016). On the other hand, according to their proponents, besides the commercial opportunities presented by a growing market of flavours, fragrances and medicines, the advantages of the approaches described above over traditional methods include guaranteeing price stability for menthol and, most importantly, the environmental sustainability of biosynthetic compounds. This is because the proposed routes do not rely on agricultural systems, utilise renewable feedstock (e.g. microorganisms, glucose) and arguably prevent negative impacts on biodiversity and the consumption of limited, rare natural resources (Chang and Keasling, 2006; Hansen et al., 2009; Toogood et al., 2015). In what follows, we take a closer look at the background narrative of the bio-turn in menthol production to flesh out the main expectations and promises associated with a transition from conventional to biosynthetic methods of production of this ubiquitous compound. The discussion presented in the next section draws on a reflection of the drivers behind the sociotechnical transition in menthol production obtained through nine semi-structured qualitative interviews conducted with scientists involved in the development of biosynthetic routes for menthol production in the UK.

## 4 Expectations and promises of the bioturn in menthol production

Along with vanillin and artemisinin, menthol is a type of terpenoid, a family of over 40,000 compounds naturally produced by plants and microorganisms. Terpenoids are involved in fundamental parts of plants' metabolism, such as photosynthesis and respiration (Aharoni et al., 2005). Because of their flavouring and scent properties they are largely used in several products at an industrial scale. However, plants tend to synthesize only small amounts of these compounds and this is especially the case of high-value terpenoid products, which could represent <3% of the total dry weight of the plant (Roberts, 2007). In this context, the nature of the biological turn in menthol production is similar to that of other sociotechnical transitions aimed at optimising and increasing production in a given techno-industrial sector. The gradual development of synthetic biology technologies could therefore be seen as “a move from naturally-sourced to laboratory-sourced molecules” in a way that can be compared to an optimisation of production in heavy technologically-driven fields such as warfare, car and plastics manufacturing (Wellhausen and Mukunda, 2009: 115–116).

However, one of the distinguishing features of emerging technologies such as those pursued by synthetic biology is that they are also driven by motivations that are in stark contrast with the outcomes of conventional sociotechnical systems of mass production supporting the industries mentioned above. That is, they are framed by promoters not only as reliable substitutes to incumbent technologies (i.e. in that they fulfil the functions of the latter), but also as solutions to societal challenges, including those created by conventional technologies themselves (e.g. environmental degradation, depletion of resources). Whether they are able to fulfil these promises is open to debate. The key point here is that the biosynthetic production of terpenoids such as menthol sits between the paradigms of “white biotechnology” and “green chemistry”. This means the ideal detachment from an oil-based economy towards a bioeconomy associated with a more public-friendly image of the chemical industry which is also more independent from fossil fuels (e.g. Clark, 2006; Lorenz and Zinke, 2005). The value of innovative, biosynthetic products is therefore framed around economic and sustainability goals, as a direct response to environmental and societal needs (see Jullesson et al., 2015; Wurtzel and Kutchan, 2016).

### 4.1 Biosynthetic menthol as challenge-led innovation

Biosynthetic menthol is a challenge-led innovation driven by concerns related to climate change, economic growth and security of resources. The potential benefits of synthetic biology menthol and the expectations that come along with them can be framed in three broad dimensions, namely a) economic reliability, b) environmental and social sustainability and c) technological efficiency.

These dimensions summarise the main motivations for the biological turn and the transition from conventional to more advanced pathways in menthol production. They belong to a particular understanding of performance, aggregating the elements that synthetic biology proponents expect to be superior to those of incumbent technologies (i.e. natural and synthetic menthol). Economic reliability refers to both the economic advantages of profiting from a high-value compound, and increased economic stability due to the emergence of a domestic and more reliable supply chain. In this regard, moving from menthol production based on mint plants farming to large-scale production of menthol in the laboratory would mean a) avoiding seasonal variation in supply, price fluctuations, and geopolitical issues that may affect supply chains; and b) increasing self-sufficiency and security of supply of a commodity that cannot be agriculturally produced in countries such as the UK.

Biosynthetic menthol is regarded as more “sustainable” and “renewable” than that produced via conventional pathways. In terms of environmental performance, the expectations around biosynthetic menthol are very much related to concerns regarding greenhouse gas (GHG) emissions and contamination from chemicals used in mint farming. Here, potential benefits are expected to derive from the reduced carbon footprint of a shorter supply chain as synthetic biologists argue that menthol is currently imported to countries in the North from countries in the South, such as India, which involves a large transportation route. Moreover, the use of chemicals such as pesticides and herbicides in the conventional production of mint are expected to be avoided by producing menthol in the laboratory, leading to less contamination of agricultural fields and release of GHG in the atmosphere.

This perception of an increased sustainability is also applied to the societal dimension of a biological turn in menthol production. The conventional production of menthol relies on mint grown in countries like India or China. A key argument indicated by scientists is that, by reducing demand of mint from importing countries such as the UK, land could become available for alternative, arguably more valuable, uses to farmers, such as growing food crops or higher value products.

Building biological factories for producing menthol in the laboratory is also expected to lead to higher efficiency and productivity in the process when considering that, differently from microorganisms such as bacteria, mint plants take considerably more time to grow. In this context, the flexibility of a lab-based production system could be much higher than agricultural production in both technical and economic terms. As explained by scientists, working on a common pathway in the laboratory gives you the option of exploring a whole range of compounds and producing something else for a different market in the near future, in case industry and consumer preferences change. Moreover, through biosynthetic production methods, menthol could be directly obtained in the laboratory as a highly pure compound, which is regarded as more desirable than following a series of resource-intensive steps for menthol purification when obtained using conventional processes such as in the case of chemically-based production. One important aspect of potentially purer menthol produced by microorganisms in the laboratory is that it is expected to avoid much of the concerns regarding potential contamination in the chemical and natural production. In this regard, the aim of those involved in its production is to be able to offer a product that can be labelled as natural (i.e. biologically, instead of chemically-produced), assumed to be perceived as superior by markets and ultimately better accepted by consumers.

## 5 Implications for anticipatory governance and RRI

Various aspects related to a sociotechnical transition from the conventional to the biosynthetic production of menthol and also the consideration of key claims and expectations embedded in such a transition have been discussed so far. We carried out an assessment of these two components of a bio-turn with the aims of understanding the potential consequences of a transition in menthol production and providing a critical appraisal of its underpinning promises. From this assessment, a series of challenges for the development of synthetic biology have emerged. They can be grouped and summarised in five dimensions, namely, social justice; environmental sustainability; infrastructure and business models; and public perception and public interest. Elaborating on these challenges and connecting them back to the discussion offered earlier in the paper (i.e. Sections 3 and 4), in this section we distil insights from the case of the bio-turn in menthol production with a view to contributing to anticipatory governance and RRI. While the challenges and implications that we highlight are grounded on the case of menthol, we suggest that there are inferences that can contribute towards outlining an agenda for the anticipatory governance and responsible development of synthetic biology, particularly in (but not limited to) the growing area of fine and specialty chemicals.

### 5.1 Social justice

Despite the potential to deliver on the environmental and societal benefits outlined by synthetic biology developers and supporters, the bio-turn in menthol production may also involve a series of social justice challenges that are worth considering in the context of an end-to-end assessment of biosynthetically produced menthol. These go beyond potential technological bottlenecks, to include economic and social side-effects of large-scale changes in menthol supply chains. The social and political side of the biological turn includes a series of considerations related to how its consequences will unfold, and whether they will be positive or negative, and for *whom*. Given the concentration of global menthol production in South Asia, the potential drawbacks of displacing menthol production from currently sourcing countries could most impact countries such as India. In this regard, the loss of an export market for these producers could lead to job losses and other negative impacts on the livelihoods of many communities directly or indirectly dependent on mint as a valuable agricultural commodity. This is especially the case for India, where mint farmers, who are small-scale producers and depend on subsistence farming, already struggle with a lack of basic infrastructure and difficulties associated with regulated marketing and support price systems (Kumar et al., 2011).

The specificities of the places where mint plants are grown, associated with the fact that most of menthol production still relies on agriculturally-derived menthol in comparison to the growing market for synthetic menthol, highlight a challenge not just for scientists and innovators, but also for industry. How can researchers and companies be ethically-minded and reflective so as to understand and prepare for the wider implications of translating science into products that can disrupt existing markets and livelihoods? The market prices of natural menthol are volatile. This is not only because of varying yields, but it is also associated with the pressure on prices exerted by competing markets. In the same way that cheaper synthetic menthol forces the price of natural menthol down, more efficient (and thus cheaper) processes of production of biosynthetic menthol could put pressure on Indian farmers. This entails an important social justice challenge related to placing the burden of changing markets on societal groups who are already vulnerable.

For anticipatory governance and RRI agendas, addressing this challenge means asking how responsibility for influencing these markets, and potentially affective the livelihoods of farmers, is attributed and allocated. For example, how should we appraise the potential indirect impacts of a radical displacement of the supply chain if biosynthetic menthol were to be produced by companies in Western, higher-income countries? How would this appraisal be different if companies established themselves in lower-income countries? And, what mechanisms are available to ameliorate, if not avoid, displacement? Anticipating the distribution of benefits and burdens of a transition from conventional to biosynthetic menthol demands investigating how potential indirect impacts unfold in a case-by-case approach, understanding how exactly markets are connected between countries and the dynamics of domestic markets for menthol.

### 5.2 Social resilience

Related to the previous challenge, there is a need to also understand the connection between social resilience and sociotechnical transitions (see Smith and Stirling, 2008). There are expectations on the part of synthetic biology scientists that those who currently rely on the agricultural production of menthol today (i.e. impoverished farmers) could find alternative uses for land or alternative markets for their products in the event that they lose their export markets. However, resilience and adaptation depends on several factors that are at the same time technical (e.g. feasibility of alternative systems), socio-economic (e.g. resources and capacity of the actors involved in the supply chain to adapt to alternative systems), cultural (i.e. alignment to people's values and concerns) and political-institutional (e.g. how domestic governments will respond with regulations, subsidies etc.) (see Adger, 2000; Obrist et al., 2010; Maclean et al., 2014). The Indian government has invested heavily over the years in developing technologies for mint production. Moreover, mint farming typically involves several family members for which the disruption in their livelihoods is greater in the absence of alternative income from one of the members. Anticipating a smooth transition to new uses of the land by Indian farmers in a potential future market that trades mostly synthetic and biosynthetic menthol, could be misleading.

This is an understudied dimension and it is difficult to establish a parallel with other target flavours for synthetic biology such as vanilla. In this case, chemically-produced synthetic vanillin dominates markets and natural vanilla represents only 1% of the global production (Hansen et al., 2009). This is a very different picture from that of menthol, where natural production is responsible for 70% of the market, as previously indicated. It is unclear what has been the impact on natural vanilla growers, which have taken part in training programs to increase the sustainability of their businesses (Braw, 2014) and whether this would be feasible at a much larger-scale for the case of menthol. The challenge of social resilience is a very relevant, yet overlooked, dimension in RRI approaches to the assessment of emerging technologies.

### 5.3 Environmental sustainability

Claims around the environmental sustainability of synthetic biology developments are widespread and are generally mobilized in the context of a transition from petrochemical-based to more sustainable, renewable bio-economies (e.g. Ye and Bhatia, 2012; Lopes, 2015). However, studies dedicated to investigating and providing evidence to the claims regarding the environmental sustainability of biosynthetic compounds are missing. Whereas biofuels, for example, enjoyed much attention from tools such as life cycle assessment (LCA) to anticipate and address some of the questions regarding their sustainability (see McManus et al., 2015), the environmental performance of other emerging bio-based chemicals has been largely overlooked (Herrgard et al., 2015). This is the case of fine chemical compounds such as biosynthetic menthol, vanillin and other targets of the flavour and fragrances industry, for which no LCA studies or similar analyses are available. Nevertheless, it is precisely the expected superior environmental performance of biosynthetic menthol that differentiates it from natural and synthetic menthol production. Indeed, the latter is typically portrayed as an environmentally *un*friendly solution to the problem of availability and price volatility of natural menthol.

There is an urgent need for prospective studies to investigate the potential environmental benefits and risks of biosynthetic value chains and compare them to incumbent natural and synthetic methods of production. Building on the lessons learned from LCAs in other fields (McManus et al., 2015), the sustainability assessment of biosynthetic compounds should be transparent about issues regarding the complexity and uncertainty of sociotechnical systems and be open to deal with value and ethical judgements. An important challenge for these prospective studies is, however, their ability to produce models that reflect a real-world, large-scale production of menthol. Biosynthetic menthol has only been produced at laboratories and scalling-up is one of the technical bottlenecks faced by this alternative production pathway.

### 5.4 Infrastructure and business models

Digitisation and automation increasingly characterise synthetic biology at all stages of product development from designing and building DNA constructs to testing biosynthetic compounds using a series of robotic systems (see Johnson et al., 2016). High infrastructural costs and highly specialised knowledge to work within digitised and automated infrastructures mean not only that a limited workforce is needed, but also that a limited number of actors are expected to play a central role in the upstream part of the value chain of biosynthetic compounds. Emerging synthetic biology companies raise large amounts of investments from key venture capitalists to support their infrastructure, including extensive use of computers, data analytics, and robots, with the hope that in the near future production costs can be lower due to optimised, automatic processes (Check-Hayden, 2015). These firms search for niche markets that are less regulated and less competitive than those like pharmaceuticals or fuel (Check-Hayden, 2015). The sector of fine chemicals for the flavour and fragrance industries, such as menthol and vanillin is one of them.

In this context, a key point relates to the kinds of business models being generated alongside burgeoning funding for synthetic biology research and innovation and efforts to translate these endeavours into real-world applications. Understanding business models as a general characterisation of the functioning of a firm and the social, environmental and economic value creation goals it pursues (Massa et al., 2016), questions for RRI include, for example, what would be considered as a responsible way for companies to generate revenues and societal benefit and how can these emerging technologies be more inclusive and address concerns of a range of different societal groups. The potential of synthetic biology to contribute to societal challenges is often emphasised as one of its greatest assets. How exactly biosynthetic methods for producing current and new compounds of interest could contribute to the public interest is, however, a less explored angle. Biosynthetic menthol has attracted the attention of science and industry because it is a ubiquitous compound with a sizeable market that entails great economic opportunities. The potential benefits to society could be easily regarded as secondary effects – a plus in the case that large-scale production becomes feasible. Nevertheless, by prioritising economic gains, market penetration and relying on automated processes, the configuration of biosynthetic menthol production could resemble that of its synthetic alternative. This suggests that biosynthetic method could resemble the existing chemical production of menthol which is dominated by large international companies.

### 5.5 Public perception and public interest

One aspect that is perceived to, alone, determine the success (or failure) of synthetic biology is that of public perception and market acceptability of its produces, at least for those involved in developing and promoting the technology. The main objective of the biosynthetic production of menthol is not only to be able to offer a product that is competitive with natural (agriculturally-based) and synthetic menthol (chemically-based), and superior in purity and quality, but that can also potentially be labelled as ‘natural’. Labelling compounds produced by synthetic biology using genetic engineering methods as ‘natural’ is a strategy targeted at consumers. Given that synthetic biology is an arm of industrial biotechnology, this strategy reflects a concern regarding previous experiences of negative public perception towards genetically-modified (GM) organisms and a fear of a sustained rejection of GM products by citizens.

As argued by Marris (2015), a “fear of the public's fear” building on the GM organisms controversial legacy has populated the minds of scientists, scientific institutions and governments. For her, this has been the background of public engagement and outreach activities related to synthetic biology and the principle motive for her criticism of scientific imaginaries of the public, often represented as a single entity which lacks the necessary knowledge to understand – thus accept – new technologies such as synthetic biology. Sociologists have explored people's everyday practices around products which are of interest to synthetic biology (Meckin and Balmer, 2017). Investigating the very case of a transition from conventional to biosynthetic menthol, they show how *publics* (in its plural form) mobilize different ways of dealing with uncertainties that are rooted in their everyday practices, and show how these are adapted to cope with the uncertainties associated with promise of novel technologies (Meckin and Balmer, 2018). If there is a take-away message from the work developed by the social scientists cited above is that RRI experiments indicate that proponents and practitioners of synthetic biology research must reflect on and internalise the complexity of publics and their values in their everyday relationship with products such as those containing menthol. But also, as stressed by Wynne (2008), RRI should pay close attention to how public concerns are imagined and indeed fabricated – typically as issues of scientific risk, as it has been historically the case of GM technology. For synthetic biology, as for other emerging technologies, questions about what are and could be the public values (Bozeman and Sarewitz, 2011) embedded and pursued by its developments should not be taken for granted.

## 6 Conclusions

Many plant-based compounds are targeted by an expanding, global synthetic biology industry focused on the drugs and food sectors (Ong, 2018). This paper highlights the importance of adding granularity to the analyses of sociotechnical transitions in the context of the anticipatory governance of emerging technologies, such as synthetic biology. This granularity is represented by a more detailed understanding of the political economy of synthetic biology and a focus on real-world applications vis-à-vis current modes of production of compounds that synthetic biology seeks to replace. As we have shown, exploring the specific sociotechnical systems associated with the production of menthol and unpacking the promissory narratives around the strengths and weaknesses of each of these alternatives, helps in outlining key areas of interest for the anticipatory governance of synthetic biology in the field of fragrances and flavours and for the synthetic biology of other fine and specialty chemicals.

Taking a sociotechnical transition in menthol production as a case, we identify and highlight five areas of interest for anticipatory governance and the responsible development of synthetic biology. These include attention to aspects of social and distributive justice; the political and geographical particularities of sociotechnical arrangements; commonplace assumptions on environmental sustainability; the implications of changing infrastructures, ways of working and business models in new modes of production; and on how publics make sense of uncertainty of new technologies in the context of their everyday practices (see Meckin and Balmer, 2017). These are areas that need explicit consideration through anticipatory governance and by RRI more generally. Moreover, they illustrate the complexity of the implications linked to relatively mundane applications of synthetic biology and should be considered when reflecting on the desirability of biosynthetic compounds destined for the food and flavours industry.

There is no single or standard response for tackling this complexity and resolving these issues. However, as a start, consideration can be given to a series of practices that could lead to greater reflexivity and open up pathways to improve our comprehension and knowledge on the challenges described above. These practices can take place within the various approaches fostered by those working on the operationalisation of RRI. At an assessment level, they include, for instance, attention to framing questions in terms of social justice aspects in technology and impact assessments. This implies opening-up assessment processes to include the perspectives of vulnerable groups who are typically excluded from expert-driven assessment processes. Alongside more reflexive and inclusive forms of problem-framing, the capabilities of those most likely to be at risk in the context of new configurations of value-chains also need to be considered to understand whether they are able to pursue strategies that would help mitigate such risks or take advantage of any new opportunities. Where the communities at risk are groups in lower-income countries who will be directly disadvantaged (even if there are net environmental benefits or economic advantages to those living elsewhere), there is an additional onus to extend efforts not only to assess potential consequences but also to realistically explore what alternate options, mitigations and development strategies could be implemented and by whom. At an organisational level, investigation of the design, functioning and potential outcomes of proposed new business models is needed to assess how these models will generate and distribute public value. These practices entail normative commitments from a range of different actors: they can be part of approaches taken by industry itself, as well as by researchers, community and developmental organisations, regulatory bodies and decision-makers.

Although discussed in the context of menthol, an often unnoticed yet ubiquitous compound in numerous products on supermarket shelves, the challenges and questions raised by this example are certainly relevant to other fields of bio-manufacturing that involve transition from agricultural or chemical-based methods to biosynthetic production models. The findings obtained suggest that the complex yet particular value chain, market and societal issues associated with specific classes of target compounds for synthetic biology demand a case-by-case type of analysis to inform synthetic biology governance. Menthol represents only one class of bio-materials produced by emerging processes in the field of synthetic biology. However, there are many bio-variations on the synthetic biology radar for replacing natural or chemical products. For example, other targets are the compounds responsible for rose fragrances, currently derived from rose petals and oils – which alongside other everyday products, tap into a renewed image for new biotechnologies focused on sustainability, cultural appeal and consumer acceptance (Zhang, 2018). In the long list of other possible compounds, target scents and flavours of synthetic biology also include patchouli, vetiver to cocoa butter, saffron, orange and grapefruit. This signals a further governance issue, in the field of regulation, of whether and how these compounds will be labelled, particularly in the food sector. Whereas synthetic biology often frames its final products as natural (Hollywood et al., 2018), it is uncertain how its addition to food as flavouring compounds will be treated by the labelling legislation including in the United States or the European Union. The labelling approach taken and whether synthetic, artificial, or natural is indicated, it is likely to influence public perception in different ways.

The insights and issues that arise from tailored case-by-case approaches, such as the one undertaken in this paper, challenge the assumption that synthetic biology can somehow be regulated and treated as a generic single class. Also called into question is the obverse proposition that synthetic biology requires no special treatment, as existing regulations will suffice. The heterogeneous implications of biosynthetic menthol suggest that “one size does not fit all” in the regulation and anticipatory governance of synthetic biology. This presents an important challenge for emerging technologies in the contemporary bio-manufacturing context, which aim for the seamless production of multiple compounds at a flexible and fast pace. At this early stage, as the menthol case illustrates, we can already envision the societal and environmental risks of travelling along a path driven primarily by synthetic biology's intrinsic technological features. An anticipatory governance approach can help in characterising the scope and scale of such risks, and – as we have argued – this approach needs to have a focus on the specifics of particular applications or application categories. This could then open up alternate pathways to reduce or mitigate, if not avoid, these risks, through RRI engagement and action involving researchers, industry, government, communities, and publics. More broadly, we argue for situated assessments of emerging technologies such as synthetic biology which consider in detail the specific complexities of sociotechnical transitions and also engage with the real-world applications and implications of emerging technologies from an early stage and as they develop. The approach and the five areas discussed in this paper contribute to an anticipatory governance framework for emerging technologies that can help in initiating these assessments, reflecting on implications, and focusing attention on where actions might be needed. In turn, this can then inform governance mechanisms and foster a deeper understanding of distributed responsibilities on the part of the various actors involved in their development and affected by their potential consequences.

## Funding

This work was supported by the Biotechnology and Biological Sciences Research Council [Grant Number BB/M017702/1] (Manchester Synthetic Biology Research Centre for Fine and Speciality Chemicals).

## Footnotes

1 http://www.intracen.org/uploadedFiles/intracenorg/Content/Exporters/Market_Data_and_Information/Market_information/Market_Insider/Essential_Oils/An%20overview%20of%20Mentha%20arvensis.pdf

2 Companies do not readily disclose their own data on menthol prices. Alternative sources include regularly updated webpages from the chemical industry in India, such as that of Prakash Chemicals (http://www.prkchemicals.com/2016.html).

3 http://flavorscientist.com/2013/04/07/theres-money-in-menthol/

4 Based on a systematic search for original research articles and granted patents conducted in early November 2017, for which the following search terms and databases were used, respectively: menthol and (“synthetic biology” or biosynthesis or “biotechnological production”) [TOPIC], on Web of Science (WoS); menthol and (“synthetic biology” or biosynthesis or “biotechnological production”) and (recombinant or “biosynthetic gene” or “synthetic DNA” or “*E. coli*” or bacteria or yeast) [FULL TEXT], on Lens. The inclusion criterion for both searches was inventions/research focused on creating recombinant microorganisms to produce menthol as a compound of interest or any of its precursors (where this capacity is explicitly mentioned) and exclusion criteria were a) inventions/research related to the production of menthol from plants or non-renewable sources (i.e. synthetic menthol); b) inventions/research related to improving the processes covered by inventions that allow for microorganisms to produce menthol.

5 Based on a systematic search for original research articles and granted patents following the same inclusion and exclusion criteria used in the search on menthol, but applied to terpenoids. Search terms used on WoS and Lens were, respectively: [biosynthesis and (terpen* or isoprenoid)] and [(heterologous NEAR pathway) or “engineered microbe*” or “metabolic engineering” or “microbial engineering”] [TOPIC]; biosynthesis and (terpen* or isoprenoid) AND “engineered microbe*” or “metabolic engineering” or “microbial engineering” [FULL TEXT].
